# Adaptation of the System for Observing Play and Recreation in Communities (SOPARC) for the Measurement of Physical Activity in Jail Settings

**DOI:** 10.3390/ijerph17010349

**Published:** 2020-01-04

**Authors:** Ricky Camplain, Travis A. Pinn, Heather J. Williamson, George Pro, Lyle Becenti, James Bret, Crystal Luna, Julie A. Baldwin

**Affiliations:** 1 Center for Health Equity Research, Northern Arizona University, Flagstaff, AZ 86011, USA; tap227@nau.edu (T.A.P.); Heather.Williamson@nau.edu (H.J.W.); George.Pro@nau.edu (G.P.); Lyle.Becenti@nau.edu (L.B.); Julie.Baldwin@nau.edu (J.A.B.); 2 Department of Health Sciences, Northern Arizona University, Flagstaff, AZ 86011, USA; 3 Department of Occupational Therapy, Northern Arizona University, Flagstaff, AZ 86011, USA; 4 Coconino County Sheriff’s Office, Flagstaff, AZ 86001, USA; jbret@coconino.az.gov (J.B.); crluna@coconino.az.gov (C.L.)

**Keywords:** physical activity, jail, incarceration, measurement

## Abstract

Over 9 million people are incarcerated in jail each year, but physical activity has not been assessed among incarcerated populations. Measuring physical activity in the jail setting is complicated as current physical activity measurement tools are not designed for use inside jail facilities. Therefore, we adapted an evidence-based physical activity measurement tool, the System for Observing Play and Recreation in Communities (SOPARC), to assess physical activity within a jail facility. SOPARC was designed to obtain observational information on physical activity of individuals. The study team created a protocol for SOPARC for use in jail facilities. Unlike the original SOPARC, access to recreation time in jail required prior scheduling. Target areas were unnecessary as recreation spaces were enclosed. The adapted SOPARC protocol for jails included start and end times, the number of individuals that attended, and recreation time users’ physical activity levels, footwear, outerwear, uniform color, and use of mobility assistive devices. The use of SOPARC in the jail setting requires adaptation to adequately capture physical activity data among incarcerated individuals. Accurately measuring physical activity among incarcerated individuals and the environment in which they are active may allow for future development and testing of physical activity interventions in jail facilities.

## 1. Introduction

Approximately 9 million Americans are incarcerated in jail each year [1,2]. Jails are short-term facilities typically housing individuals awaiting trial or serving a sentence less than one year. Although jails may allow incarcerated individuals recreation time (structured time dedicated for recreational physical activity), it is often the only time dedicated for physical activity. Previous research indicates that most individuals incarcerated in jail do not regularly attend recreation time [3]. It is well established that physical activity can have immediate impacts on overall health [4]. For example, a 30 min bout of moderate-to-vigorous physical activity will reduce blood pressure, [4,5] improve sleep quality [4,6], and reduce symptoms of anxiety and depression [4,7]. Other benefits, such as disease risk reduction and physical function, accrue within days after adopting a new physical activity routine [4,8]. These benefits occur primarily through regulation of body temperature, adrenal activity, and neurotransmission of noradrenaline and dopamine [9] and can contribute to short-term calming effects, stress adaptation, and improved mood. This is important as health needs of individuals incarcerated in jail are complex. Individuals incarcerated in jail facilities experience high rates of hypertension [10,11,12], poor sleep quality [13], anxiety and depression [14], and more may experience anxiety-like symptoms from being incarcerated. However, 76% of individuals incarcerated in jail do not use recreation time regularly due to motivational, equipment, and safety factors [3], and physical activity levels during incarceration are unknown.

Measuring physical activity in the jail setting is difficult for multiple reasons. Researchers may not be able to use accelerometers and other physical activity measurement devices as they may be considered contraband inside jail facilities. Additionally, physical activity questionnaires are not designed for use inside jail and are subject to bias [15]. Physical activity in jail may be specific to that environment, and the numerous questionnaires available to measure physical activity [16] often include examples of physical activities that are not possible in jail (e.g., bicycling), limiting the potential for use in the jail population. Finally, jails are short-term in nature, and the median length of stay is 2 days (range 1–3381 days) [17]. The short length of stay indicates the number of individuals who attend recreation time and their physical activity levels may change frequently. Thus, an objective tool for quantifying physical activity in the jail environment is necessary.

The System for Observing Play and Recreation in Communicates (SOPARC) was designed to obtain observational information on physical activity in “open” environments, such as community parks, including relevant concurrent characteristics of the environment and its users [18]. SOPARC was designed to obtain observational, objective data on the number of participants and their physical activity levels in community environments and has been used and adapted in multiple settings [19]. During a scan, the activity of everyone is mechanically coded as sedentary (i.e., lying down, sitting, or standing), walking, or vigorous (very active). Summary counts describe the number of participants and activity modes and levels. The instrument permits physical activity level comparisons to be made among different environments or within the same setting over different time periods [18,19].

Given SOPARC’s proven ability to obtain reliable observational physical activity information [18,19,20], the potential for physical activity to impact health and wellness among incarcerated individuals, and the lack of measured physical activity in jail settings, this project sought to test a new strategy of applying SOPARC in jails and adequately measure the level of physical activity of incarcerated individuals. Our objective was to adapt the original SOPARC protocol and materials for use in the jail setting, specifically for recreation time at the Coconino County Detention Facility in Flagstaff, Arizona.

## 2. Materials and Methods

### 2.1. Study Setting

The Coconino County Detention Facility is a regional holding facility in Flagstaff, AZ, that houses a daily average of 450 individuals [21]. The detention facility consists of four subsections containing 21 housing units (dorms). Dorms are segmented by gender (male and female), by internal risk assessment (low, medium, and high security), and by known conflicts among individuals housed in the facility. Most individuals incarcerated at Coconino County Detention Facility are between the ages of 18 and 34 years and male. Additionally, most individuals incarcerated at Coconino County Detention Facility are white or American Indian/Alaska Native [17].

**Figure 1:**
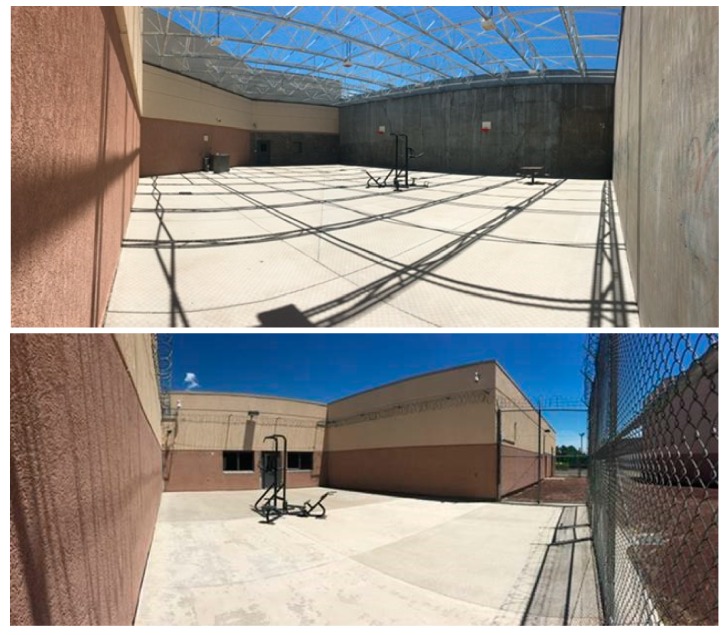
Recreation areas at Coconino County Detention Facility.

Coconino County Detention Facility offers recreation time to individuals housed in minimum to maximum security dorms. Recreation time is not always offered in jail facilities and is described by the jail administration as a privilege offered to everyone by Coconino County Detention Facility. Each of the four pods have their own recreation area (space dedicated for recreation time) with a concrete floor and equipped with a bolted down piece of exercise equipment (Figure 1). Larger recreation areas have a toilet and picnic table. One recreation area that is available to juveniles housed at Coconino County Detention facility is also equipped with basketball hoops. To note, incarcerated adults are not allowed access to basketballs; however, individuals are offered a handball for use during recreation time. Each dorm is allocated prespecified, scheduled recreation times one hour per day, five times per week. Individuals from different dorms are not allowed to be at recreation time together.

The Northern Arizona University Institutional Review Board approved this study, which included an external review by a Prison Advocate. The Office for Human Research Protections, which provides protection of the rights, welfare, and wellbeing of human subjects involved in research supported by the US Department of Health and Human Services, approved this study.

### 2.2. The System for Observing Play and Recreation in Communities (SOPARC) Adaptation

In September 2019, our team, in collaboration with Coconino County Detention Facility staff, adapted the SOPARC protocol and the SOPARC observation form to account for the unique jail setting. After meeting with jail staff regarding the day-to-day schedule of the facility, obtaining a recreation time schedule, and touring the facility’s recreation areas, the study team created a step-by-step protocol for SOPARC for use in jail facilities.

### 2.3. Training

In preparation to use the adapted SOPARC methodology and observation forms, observers attended a one-day workshop using the original SOPARC training materials available through the Active Living Research Website (https://activelivingresearch.org/soparc-system-observing-play-and-recreation-communities).

Training included lectures with definitions, instrument notation, coding conventions, and how to differentiate between the various physical activity levels. Observers practiced coding and received feedback on their scoring of videotaped samples of physical activities. Following the training videos, all researchers that conducted SOPARC observations inside the Coconino County Detention Facility attended a field-based observational training inside the facility during two scheduled recreation times using the adapted form in real-time. Prior to any data collection inside the Coconino County Detention Facility, observers also completed a jail-sponsored training available to detention facility volunteers facilitated by the Programs Coordinator and detention officers. Training included safety procedures and regulations within the Coconino County Detention Facility.

### 2.4. Observation Areas

Recreation areas were described, photographed, documented, and assessed for observation areas (area for certified assessors to observe individuals at recreation time) prior to SOPARC adaptation and observations. Observation areas are akin to SOPARC’s original “Target Areas”, comprising all potential areas for leisure time physical activity in each park or location. However, the recreation areas in the Coconino County Detention Facility are enclosed, and only one observation area (i.e., recreational space) is available to observe. The team observed two recreational spaces that were regularly used by the dorms assigned to this project. Each recreation area is fully visible from the observation area and accessible to observers, and observations were made through a two-way mirror or window.

### 2.5. Observation Preparation

Unlike the original SOPARC that was tested and used in public park spaces, access to recreation time at Coconino County Detention Facility required prior scheduling. Prior to observations, observers obtained a weekly recreation time schedule from jail personnel for the dorms that were going to be observed at recreation time. Observers then sent a schedule of observations to the Coconino County Detention Facility’s Programs Officer to be added to the facility’s weekly programming schedule. Observers were required to arrive at Coconino County Detention Facility 15 min prior to the start time of the first recreation time that was observed. Observers checked in at the front desk, and a Detention Officer escorted them to the dedicated observation area prior to the start of recreation time.

### 2.6. Recording Procedures

**Figure 2:**
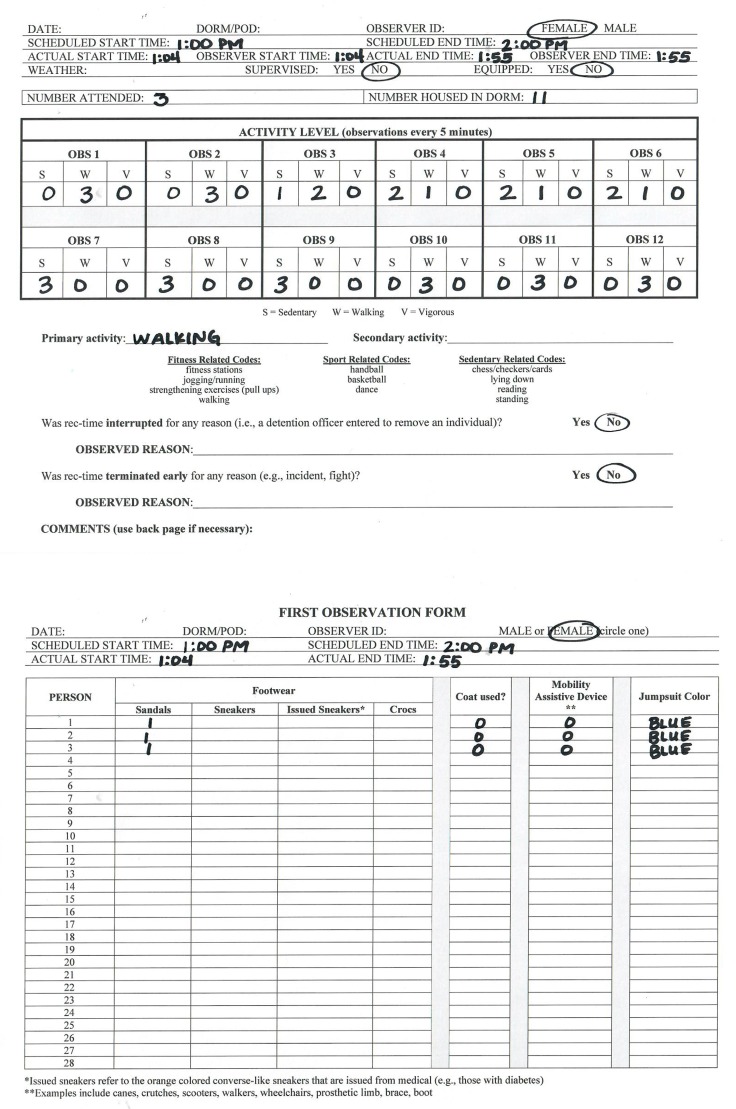
Sample SOPARC data collection form.

Prior to the start of the observation period and using the observation form (Figure 2), observers entered the scheduled date, predetermined dorm, individual observer ID, whether it was a male or female dorm, scheduled start time, scheduled end time, and a description of the weather. When individuals were escorted to recreation time, observers entered the actual start time and scanned the recreation area to count the number attended. Information regarding everyone’s footwear (individuals are issued sandals when booked into the detention facility or can purchase sneakers at commissary), outerwear (whether they were wearing an issued coat), if they were using a mobility assistive device (e.g., crutches, wheelchair), and their jumpsuit color (to determine work status) was recorded. Footwear, outerwear, the use of a mobility assistive device, and whether or not an individual has an assigned job while incarcerated may influence an individual’s recreation time attendance and physical activity and is important to capture. After the initial count, observers scanned the recreation area from left to right, simultaneously and at the same pace. During scans, observers recorded everyone’s activity level (sedentary, walking, or vigorous), according to the original SOPARC protocol. To allow for evaluation of activity over the course of recreation time, observations to record activity level were repeated every 5 min and recorded in the “Activity Level” table on the observation form (Figure 2) until recreation time was over and individuals were escorted back to their dorm. During the scheduled hour of recreation time, observers also recorded what the primary and secondary activities were in the recreation area (e.g., walking, sitting, handball). When recreation time was completed and a detention officer escorted individuals back to their dorm, observers entered the actual end time. When completed, observers asked a detention officer or other staff member for the total number of individuals housed in the dorm that was observed at recreation time. Finally, a detention officer escorted observers back to the detention facility lobby.

If no one attended the scheduled observed recreation time, observers entered the scheduled date, predetermined dorm, individual observer ID, whether it was a male or female dorm, scheduled start time, scheduled end time, and a description of the weather. Observers would then confirm with a detention officer that none of the individuals residing in the dorm assigned to the specific recreation time wanted to participate in recreation time and enter the total number attended (zero) and the total number of individuals housed in the dorm. If recreation time was cancelled for any reason, observers noted the date, time, and dorm, and the observation was rescheduled.

### 2.7. Intended Data Analysis

Physical activity measures will be counted for those engaged in sedentary, walking, and vigorous physical activity, and a proportion of individuals in each physical activity level will be calculated. We will additionally estimate the proportion of individuals who engage in moderate-to-vigorous physical activity by summing the number of individuals engaged in walking and vigorous physical activity and dividing by the total number of individuals who attended recreation time.

### 2.8. Reliability

Reliability data for physical activity codes were collected during all observation periods using pairs of assessors who made simultaneous and independent observations. Data from 489 scans were used in the reliability analysis. Observers classified each individual into one of three activity levels (sedentary, walking, vigorous). We calculated the overall proportion of agreement. Our proportion of agreement for physical activity level was 94.5%.

### 2.9. Validity

Original SOPARC activity codes, which were used in the present study, have been used in other observation systems, including Behaviors of Eating and Activity for Children’s Health: Evaluation System (BEACHES) [22], the System for Observing Fitness Instruction Time (SOFIT) [23], and the System for Observing Play and Leisure Activity in Youth (SOPLAY) [24]. Construct validity of activity codes has been established through heart rate monitoring with children 4–18 years [22,25] and with accelerometers in schools [26]. We did not determine validity of the physical activity codes in our study.

## 3. Results

Multiple changes to the original SOPARC protocol and observation form were necessary for use inside the jail setting. Table 1 includes a summary of adaptations and changes to the SOPARC with corresponding justifications for these modifications. Overall, our adaptations were necessary to account for the unique observation setting and specific goals of our overall research, which was to understand physical activity among incarcerated populations. Some adjustments and adaptations to the protocol and observation forms were based on field-based observation training and study team discussions.

Unlike the original SOPARC, access to recreation time in jail required prior scheduling using a weekly schedule. Jail staff provided observers an established weekly recreation time schedule. Each dorm selected for observation was observed once for each day of the week that recreation time was scheduled. For example, a housing unit may be observed on a Sunday at 1:00 PM, a Tuesday at 8:00 PM, a Wednesday at 1:00 PM, a Thursday at 11:00 AM, and a Saturday at 11:00 AM, not necessarily all in the same week.

Target geographic areas were also unnecessary for the protocol in the jail as recreation spaces were enclosed and only one observation area was available for observers during scheduled observations. Similar to the original SOPARC, each recreation area was visited prior to observations and was photographed, described, documented, and assessed for observation areas.

Changes made to the SOPARC observation form were also necessary. For use during recreation time in the Coconino County Detention Facility, researchers removed the “conditions for target areas” as it was not applicable to the jail setting. The recreation areas inside the jail are controlled environments, and recreation time occurs in the same location, at the same times, each day. “Ethnicity” and “age group” information was also removed from the form and protocol as both may be unreliable measures through observation. Activity-related codes (e.g., basketball, soccer) were also removed from the form, as adults participating in recreation time are not allowed these types of activities.

Scheduled and actual start and end times were added to the form to assess for how long recreation time was scheduled, and for how long individuals were at recreation time. This information is necessary to capture the formal time allotted for individuals incarcerated in jail to be physically active. To understand the proportion of individuals who attend recreation time from those allowed to attend, “number attended” and “number housed in dorm” were collected. A “weather” section was also added to provide information to help researchers assess weather-related barriers to recreation time use and physical activity (i.e., temperature, cloud cover). To allow for evaluation of physical activity over time, observers recorded activity level every 5 min during recreation time. Because jail staff control recreation time and interruptions and termination of recreation time may affect the length of time individuals can participate in recreation time, “Was recreation time interrupted or terminated early for any reason?” was added to the observation form. Finally, after removal of “ethnicity” and “age group”, “footwear”, “mobility assistive device”, “coat used”, and “jumpsuit color” were added to describe recreation time attendees.

Further adjustments made to the observation form included adding a “Female or Male” section rather than having observers count the number of females and males participating in recreation time. Jail facilities separate females and males and do not allow them to interact. An extended comment section was also added to collect further qualitative, context-specific information that may not be captured in the other sections of the form. This allows observers to note physical activity between observations, interactions between jail staff and incarcerated individuals, non-physical activities, and other relevant physical activity information. These notes will inform further adaptation of the protocol and observation form. They were also used as part of the development of questionnaires and focus group guides to further investigate physical activity among incarcerated individuals at the Coconino County Detention Facility.

## 4. Discussion

After and while the team completed observations, researchers discussed the effectiveness of the adapted SOPARC protocol and observation form. In general, the protocol and observation form were found to work well in the setting, especially after further adjustments were made after the training period. Adaptation of SOPARC for the jail setting was imperative to estimating physical activity among individuals incarcerated in jail. Few studies have measured physical activity among individuals incarcerated in prison, long-term facilities housing individuals sentenced to ≥365 days, and none, to our knowledge, have estimated physical activity in US jails. Implications regarding physical activity among individuals incarcerated in jail based on studies conducted in prison is not appropriate.

Measuring physical activity in the jail setting is difficult due to measurement issues and nonvalidated questionnaires. Of the physical activity research done in prison settings, most use questionnaires not validated among correctional populations [27,28,29], while others do not indicate how physical activity was measured [30]. When estimating physical activity among correctional populations, it is imperative to measure with a high degree of validity, and the International Physical Activity Questionnaire, a common instrument in the aforementioned studies, found that the majority of validation studies of the instrument found little correlation with objective measures [31]. Additionally, the short-term nature of jail facilities [17] makes it difficult to recruit individuals for data collection, a limitation of which the adaptation of SOPARC presented in this study eliminates.

Through the adaptation process, we found unique problems to the Coconino County Detention Facility. In one of the two observation areas, the two-way mirror did not seem to shield observers from being seen by the incarcerated individuals during night-time observations. To limit observation effect during night observations, observers positioned themselves in the observation area to be less visible and did not observe recreation time too closely between official scans (e.g., not continually standing close to the two-way mirror or turned away from the recreation area). Observers were also not escorted to the observation area until after the individuals were escorted to recreation time as the observation areas were in the path from the dorms to the recreation areas.

Another common reflection was related to visibility difficulties. Because observers had limited visibility as a result of small windows, observers had to step very close to the windows or further back in a central area during official scans to maximize the number of recreation time users that could be observed in one scan. This was a limitation of the jail setting. Though it did not happen often, some recreation time users were not visible during observation periods. When an individual could not be observed through the window, they were not included in the observation.

Another reflection about the SOPARC adaptation was related to accessibility of observation areas and recreation time by observers. In some instances, staff escorted the observers to recreation time after it started. There were also scheduled observations in which observers were not allowed into the jail or a staff member was not available to escort observers into the facility. When an observation did not take place due to conflicts or cancellations, the specific dorm, day, and time were rescheduled. There were also occurrences when jail staff did not know the reasons observers were in the facility and therefore did not know how they could help observers. Observers must always be escorted; thus, timing is not always seamless, especially if the officer has other more pressing tasks.

Other limitations to the SOPARC adaptation for jail settings are also of note. Through systematic observation, observers do not interact with individuals at recreation time and thus have little context about the individual attendees other than what can be directly observed. Observers also cannot observe those incarcerated in the facility who did not attend recreation time. Thus, observers cannot capture physical activity that may happen outside of designated recreation times, including in their dorms or any work-related activities (e.g., cleaning). Through direct observation, we also cannot determine reasons why individuals chose to attend recreation. Future, formative research should include focus groups and interviews with individuals incarcerated in jail to determine barriers and facilitators to attending recreation time and being physically active while incarcerated. Qualitative research will compliment any objective findings to help develop physical activity interventions in jail facilities.

## 5. Conclusions

Measuring physical activity among individuals incarcerated in jail is a challenging research endeavor. Traditional methods of measurement, including surveys and accelerometers, are typically not allowed inside jails. To understand physical activity in jail, we must first develop a reliable method of collecting physical activity-related information. We sought to adapt SOPARC, a systematic observation form to capture physical activity among individuals in a setting designated for leisure and play, to use among incarcerated populations. Although similar to the original SOPARC protocol, the use of SOPARC in the jail setting required adaptation to adequately capture observational data. Physical activity among individuals incarcerated in jail may improve health conditions. Accurately measuring physical activity among incarcerated individuals and considering the environment in which they are active may allow for future development and testing of physical activity interventions in jail facilities.

## Figures and Tables

**Table 1:** Description of adaptations and changes from original System of Observing Play and Recreation in Communities (SOPARC) observation form for use in the correctional setting.

	Adaptations and Changes	Justification
Form Items Removed	Limited “Conditions for target area”	Accessible, useable, organized, dark, and empty not necessary because these are controlled environments. Rec-time occurs in the same location each time, so this was not applicable to the jail setting.
	Removed “Ethnicity”	Unreliable to measure through observation.
	Removed “age group”	Unreliable to measure through observation.
	Limited activity-related codes	There are a limited number of activities that incarcerated rec-time users can engage in.
Form Items Added	Added “number attended” and “number housed in dorm” sections	Provides information that allows team members to assess proportion of housing unit residents who use rec-time.
	Added “weather” section	Provides information that can help team members assess barrier to physical activity (e.g., cold weather or hot weather).
	Multiple activity level observations	The rec-time spaces are singular and closed. By observing physical activity periodically during a single rec-time, our team was able to get better assess patterns.
	Added “interrupted” and “stopped early” sections	Provides information about jail-specific patterns and potential obstacles to physical activity.
	Added “footwear”, “mobility device” and “coat used” on a secondary form to be completed once per hour of rec-time	Provides information that can help team members assess barriers to physical activity. This was added on a second page, because it would not fit on a single page.
Form Adjustments	“Female or Male” was added.	Because correctional facilities either separate females and males and do not allow them to interact or are sex-specific, a “Female or Male” section was included rather than determining the gender of each recreation time participant.
	Comment section moved to the back of the page.	We extended the comment section to add qualitative data to our systemic observations. This allowed us to gather other relevant details not on the form.
